# Uptake and adherence of a self-directed internet-based mental health intervention with tailored e-mail reminders in senior high schools in Norway

**DOI:** 10.1186/1471-244X-14-14

**Published:** 2014-01-21

**Authors:** Kjersti R Lillevoll, Hans Christian B Vangberg, Kathleen M Griffiths, Knut Waterloo, Martin R Eisemann

**Affiliations:** 1 Department of Psychology, University of Tromsø, Tromsø, Norway; 2 Centre for Mental Health Research, The Australian National University, Canberra, Australia

**Keywords:** Internet-based cognitive behavioural therapy, Depression, Adolescents, E-mail support

## Abstract

**Background:**

Internet-based cognitive behavioural therapy (ICBT) is a promising approach to the prevention and reduction of depressive symptoms among adolescents. This study aimed to evaluate the feasibility and efficacy of disseminating a self-directed internet-based mental health intervention (MoodGYM) in senior high schools. It also sought to investigate possible effects of tailored and weekly e-mail reminders on initial uptake and adherence to the intervention.

**Method:**

A baseline survey was conducted in four senior high schools in two Norwegian municipalities (n = 1337). 52.8% (707/1337) of the students consented to further participation in the trial and were randomly allocated to one of three MoodGYM intervention groups (tailored weekly e-mail reminder (n = 175), standardized weekly e-mail reminder (n = 176 ) or no e-mail reminder (n = 175)) or a waitlist control group (n = 180). We tested for effects of the intervention on depression and self-esteem using multivariate analysis of variance, effects of tailored e-mail and self-reported current need of help on initial uptake of the intervention using logistic regression and the effect of weekly e-mails on adherence using ordinal regression.

**Results:**

There was substantial non-participation from the intervention, with only 8.5% (45/527) participants logging on to MoodGYM, and few proceeding beyond the first part of the programme. No significant effect on depression or self-esteem was found among the sample as a whole or among participants with elevated depression scores at baseline. Having a higher average grade in senior high school predicted initial uptake of the intervention, but tailored e-mail and self-reported current need of help did not. Weekly e-mail prompts did not predict adherence. The main reasons for non-use reported were lack of time/forgetting about it and doubt about the usefulness of the program.

**Conclusion:**

Overall, disseminating a self-directed internet-based intervention to a school population proved difficult despite steps taken to reduce barriers in terms of tailoring feedback and dispatching weekly e-mail reminders. Providing mental health interventions within the school environment is likely to ensure better uptake among senior high school students, but there is a need to effectively communicate that such programmes can be helpful.

**Trial registration:**

The trial was registered retrospectively as ACTRN12612001106820.

## Background

Depression among adolescents has been recognized as a significant mental health problem, with point prevalence estimates varying between 1% and 6% [1,2]. Longitudinal studies of community samples estimate a lifetime prevalence of clinical depression up to late adolescence and early adulthood between 4% and 25%. The recurrent nature of depression points to the importance of prevention and early intervention among this group [3].

The majority of adolescents suffering from depression remain untreated [4-6]. Several barriers to help-seeking for common mental health problems have been identified [7-9]. The most prominent include stigma and embarrassment about seeking help, confidentiality concerns and poor mental health literacy and the belief that one should handle problems without outside help (self-reliance).

Using the Internet to offer mental health interventions to the population is a promising approach to overcoming many of these barriers to help-seeking among adolescents, such as stigma, confidentiality concerns and self-reliance. The positive effect of internet-based cognitive behavioural therapy (ICBT) on depression among adults has been documented in randomized controlled trials [10-14] and community populations [15,16]. Although ICBT with therapist support shows larger effect sizes, fully automated, unguided interventions are effective in reducing depressive symptoms [17]. Furthermore, programs aiming to enhance skills on the individual level, such as promoting healthy cognitive and behavioural patterns, may be one means of preventing depression also in currently non-depressed persons [16,18]. The evidence for effectiveness of computerized or internet-based CBT for adolescents and young adults is emerging, with a number of studies reporting that such interventions are effective in preventing or reducing depressive symptoms in adolescents and young adults [19-22] and in schools in particular [23-25]. For example, there is evidence from an Australian study that iCBT can prevent new cases of depression in boys and anxiety in both boys and girls when delivered to students without symptoms in the classroom [cite Calear et al.] This raises the question of whether fully automated ICBT outside the classroom might also be effective as a universal intervention to adolescents in schools, as a mental health promotion tool for the general adolescent population and prevention or early intervention for persons with elevated levels of depressive symptoms.

One factor that may prevent the uptake of fully automated ICBT in individuals with elevated symptoms is poor mental health literacy; for example, having difficulties identifying symptoms of depression. It has been proposed that providing feedback about an individual’s level of depression and anxiety might facilitate engagement with e-health applications [26]. Increasing the awareness of symptom level among individuals at risk of depression could be an effective way of promoting uptake of ICBT for depression. This could be achieved through tailoring feedback based on targeted individual factors. The latter might not only include symptom level but also other factors associated with help-seeking behaviours [9]. Tailoring interventions or feedback are already utilized in life style or health related interventions [27-29] and are emerging within the field of depression [30]. An initial screening including a measure of depression could form the basis for such feedback and thus encourage uptake of the ICBT among adolescents with elevated symptom levels. Adolescents with no depressive symptoms or mental health complaints may have poor motivation to enrol in a mental health promotion program, but a positive framing of the intervention and focus on strengthening protective factors could enhance interest. The cognitive vulnerability model of depression proposes that low self-esteem increases risk of depression, and has received empirical support [31,32]. Thus, this is a relevant factor to address in mental health promotion programs.

An issue considered as a barrier to the implementation of Internet interventions in general, is the poor adherence associated with open access websites compared to monitored settings such as randomized controlled trials [33,34]. The use of "push" factors or reminders has been recommended by several researchers [20,35-37]. Research has yielded encouraging results concerning the effect of reminders (post card, e-mail, telephone or instant messaging prompts) on adherence. Overall, findings suggest that adding reminders encourages sustained usage of intervention web sites [38-42]. However, the results are not unambiguous [43,44]. Further, to our knowledge, the possible benefit of tailored e-mail reminders supporting a self-directed mental health intervention in an adolescent sample has yet to be investigated.

### Hypothesis

The overall purpose of the study was to explore the feasibility of disseminating a fully automated, self-directed internet-based mental health intervention (MoodGYM) in high schools. We aimed to test the efficacy of the intervention in reducing depressive symptoms and increasing self-esteem in the sample as a whole, and more specifically in participants with elevated levels of symptoms of depression. We hypothesized that there would be:

1. Reduction of depressive symptoms and increase in self-esteem in the sample as a whole (prevention hypothesis).

2. Reduction of depressive symptoms in participants with elevated scores at baseline (treatment hypothesis).

Furthermore, we aimed to investigate whether e-mail tailoring in terms of providing participants with relevant feedback, would enhance initial uptake of MoodGYM among students reporting a need for help with psychosocial problems. Further, we aimed to investigate the relationship between weekly reminders and adherence to MoodGYM.

We hypothesized that there would be:

3. Better initial uptake (more participants commencing MoodGYM) among students with a reported current need of help in the tailored e-mail group compared to the group not receiving tailored e-mails.

4. Better adherence (participants persisting in MoodGYM use for a longer period) in the groups receiving weekly reminders.

## Method

### Design

The study involved a four-arm randomized controlled trial with measures administered at baseline and at post- intervention at 6–7 weeks following the commencement of the intervention. Participants were recruited from four different senior high schools in Troms county in Northern Norway. The schools were not selected at random; rather, the school administration volunteered for participation in the MoodGYM trial. All schools in the county were offered the opportunity to enter the study, but several schools were already participating in alternative interventions or declined for other reasons.

The study was conducted from September to November 2009. It was approved by the Norwegian Regional Ethics Committee North (REK NORD 114–2006). According to Norwegian legislations, individuals between the ages of 16 and 18 years can decide without their parents’ consent whether to enter non-intrusive research projects (Norwegian Health Research Act of 2008 [45]). Individuals under the age of 16 need parental consent, but if the adolescent does not wish to disclose information to parents, the legislation indicates that this should be respected. A proportion of students had not yet reached the age of 16 when entering senior high school, but was scheduled to do so within the school term. Since treating students under the age of 16 differently by requesting parental consent might have represented an obstacle to the adolescents, no parental consent was requested. Parents did however receive information about the study from the schools. The Regional Ethics Committee approved the use of the data from participants under the age of 16.

### The internet-based intervention

The intervention in the trial was MoodGYM, a cognitive behavioural therapy based interactive program consisting of five modules and a personal workbook [46]. It is designed to prevent and reduce depressive symptoms in young people. Each module has a specific theme and is designed to take between 30–45 minutes to complete. The first module introduces “characters” that model thinking patterns recognizable to the user. It further demonstrates the interaction between mood and the way of thinking using animated diagrams and interactive exercises. Module two describes different types of dysfunctional thinking and how to challenge the validity of negative thoughts. It also provides a self-assessment for dysfunctional thought. Module three presents the user with several strategies for overcoming dysfunctional thoughts and also assesses self-esteem to provide training to increase it. The next module, number four, looks at life-event stress, pleasant events and activities in order to increase focus upon the activities creating more positive experiences and emotions. The final and fifth module covers problems concerning typical issues regarding relationship break-ups. Exercises from the workbook are integrated into each of the above modules. Every module is designed to be completed in approximately 45–60 minutes, and in the latest version of the MoodGYM program (Mark III) core assessments are compulsory not allowing the user to skip or alternate between the different modules.

### Participant recruitment

Participants were recruited through visits of the research group to the four participating schools. The recruitment process included the delivery of a short lecture about mental health in general, and a presentation on MoodGYM in particular, followed by an invitation to the students to participate in a study of MoodGYM. Each participant in the study signed a written consent form at the day of the information session and received a unique identification number to be entered in the baseline and post intervention questionnaire. Students consenting to enter the study could either, depending on their preference, choose to participate in the baseline survey only, or in the MoodGYM trial, as well. As an incentive to participate, the id-number of all students completing the baseline survey was entered into a lottery with a chance to win an iPod. For those consenting to participate in the MoodGYM trial, a second lottery with an iPod prize was arranged.

## Measures

### Participant characteristics

Demographic characteristics including gender, age and average grade in high school were collected as were self-reported current and previous need of help for psychological problems and use of mental health services.

### Depressive symptoms

Level of depression was measured using a Norwegian version of the Centre for Epidemiologic Studies Depression scale (CES-D) designed to measure depressive symptomatology in the general population [47]. Responses are rated on a four-point scale. Scores range from 0 to 60, where scores 16 or higher are regarded as reflecting a clinical level of depression. However, due to developmental factors in adolescent samples the CES-D score may be inflated [48]. A cut-off score above 24 has therefore been suggested to detect clinically diagnosable cases [49]. The Norwegian version of this scale has previously been used in studies with satisfactory internal consistency [50-52]. The Cronbach alpha for the current study was .88. The tailored e-mails differentiated between low risk of depression (CES-D sum score < 16), moderate risk (16–24) and high risk (> 24).

### Self-efficacy

Self-efficacy was assessed using the Norwegian version of the General Self-Efficacy Scale (GSE) [53]. The scale consists of 10 items and is designed to assess the individual’s belief in their ability to handle difficult situations in an appropriate way. Responses are reported on a four-point scale ranging from “not at all true” to “exactly true”. The psychometric properties of the Norwegian version of this scale has been found satisfactory [54]. The Cronbach alpha for the current study was .88. Individually tailored e-mails in this study differentiated between individuals with low (sum score < 30) and high (30 or more) self-efficacy.

### Self-esteem

Self-esteem was assessed using the Norwegian version of the Rosenberg Self Esteem Scale (RSES) [55] as a measure of global self-esteem. The scale consists of 10 statements related to overall feelings of self-worth or self-acceptance. The items are answered on a four-point scale ranging from strongly agree (1) to strongly disagree (4) yielding a score between 10 and 40. The Norwegian version of the scale has yielded satisfactory psychometric properties [56]. The Cronbach alpha obtained in the current study was .88. Individually tailored e-mails in this study differentiated between individuals with low (sum score < 25) and high (25 or more) self-esteem.

### Reasons for non-use

The post-intervention questionnaire included questions regarding reasons for non-use of the intervention.

### Intervention study procedure

The baseline survey was undertaken on the day of the information session at the school. Participants either completed the survey online (56.7%), or on paper (43.3%). Participants who consented to participate in the trial were randomly allocated to either a control group or one of three intervention groups with or without reminders. The randomization was undertaken using the SPSS to generate random numbers, which then were ordered in ascending order and allocated numbers from 1–4. This was undertaken by the first author. Participants allocated to the MoodGYM trial intervention groups received an e-mail within one week of completing the baseline survey at their school, containing their user name and password for the internet program.

The intervention was undertaken during the participant’s own time. Except for the use of the automated e-mails in two conditions, the ICBT-program delivery was unguided. Progress through MoodGYM was tracked on and later retrieved from a server at the Australian National University (ANU). Six weeks after the pre-test the researchers returned to the school to collect the post-intervention data.

Following completion of the post-intervention phase, the researchers debriefed the students about the study design and explained to participants in the control group how sign up to undertake the MoodGYM program.

### Intervention groups in the MoodGYM trial

Participants were allocated into either a control group (group 1; n = 180) or one of the three intervention groups: no reminders (group 2; n = 176), standard e-mail reminders (group 3; n = 176) or tailored e-mail reminders (group 4; n = 175).

The content of each of the interventions was as follows:

Group 1. The control group received an e-mail within one week after the baseline survey informing them that they were enrolled in the study and would receive further information. After the post-test all students were informed that they had been part of the control group, and about how to obtain access to MoodGYM.

Group 2. MoodGYM without reminders. This group received an e-mail within one week after the baseline survey with user name and password to register in MoodGYM. They were instructed to complete one module of MoodGYM per week.

Group 3. MoodGYM with standard reminders. The same content as Group 2 and standard e-mails preceding each module providing a general introduction to the topic of each module.

Group 4. MoodGYM with tailored reminders. The same content as Group 2 and individually tailored e-mails preceding each module. The tailored information was based on data collected in the baseline survey on risk of depression, level of self-efficacy and self-esteem. These variables were selected based on their interrelatedness and relevance to help-seeking behaviors [57-59]. The first tailored message included the standard general introduction to module one, as well as feedback on the individual risk of depression based on the CES-D; the second message included the standard general introduction to module two as well as information about their current level of self-esteem; the third message provided information concerning the content of module three in MoodGYM adapted to their level of depression; the fourth message provided feedback regarding their level of self-efficacy which was related to the content of module four; the fifth message introduced the topic of module five adapted to their level of self-esteem. The e-mails to participants with low levels of depressive symptoms, high self-esteem and self-efficacy were tailored to encourage use of MoodGYM in order to learn more about mental health problems.

The e-mails were automatically generated by software separate from the MoodGYM program, with all participants in groups 3 and 4 receiving weekly e-mails regardless of their level of MoodGYM use. The first author undertook the process of registering participants in the e-mail software and ensuring the weekly e-mail dispatch.

### Statistical analysis

Data analyses were performed using IBM SPSS statistics 19 for Windows. We allowed for 10% missing values on the CES-D, RSES and GSE scales, replacing them with mean values.

Chi square tests and one-way analyses of variance (ANOVA) were conducted to assess whether the MoodGYM trial participants differed in demographic characteristics and depressive symptoms, self-esteem and self-efficacy from the larger sample of students who participated in the baseline survey only. Intra-class correlations for each year (year 1, 2 and 3) within each school were calculated to assess dependence in the data.

To test hypothesis one, whether there was a reduction in depressive symptoms and an increase in self-esteem in the sample as a whole, a multivariate analysis of variance was conducted. The independent variable was MoodGYM use (yes/no).

To test hypothesis two, whether there was a reduction of depressive symptoms in participants with elevated scores (CES-D > 16) we conducted a multivariate analysis of variance. We also tested if there was an increase in self-esteem in this group. The independent variable was MoodGYM use (yes/no).

To test hypothesis three, whether a self-reported current need of help and tailored e-mail reminders predict uptake, a logistic regression analysis was performed using the independent variables current need of help, intervention group and the interaction variable with current need and group. Uptake was defined as use or non-use of MoodGYM.

To test hypothesis four, whether weekly e-mail reminders increase adherence, an ordinal regression analysis was conducted with weekly reminders as the independent variable. Adherence was measured as number of modules with 25% progression or more, with modules 2–5 collapsed to one category to increase power. Accordingly, adherence was divided into categories of non-participation, one module or two or more modules.

Previous studies of internet-based self-help for depression and anxiety have shown effect-sizes ranging from 0.00 to 0.90 both for depression and anxiety measures. Effect-size of 0.6 at post-test was estimated. To test for intergroup differences of this magnitude by analysis of variance at a 0.05 level and with a power of 0.80, a sample of 45 in each condition is needed. To allow for an average attrition rate of 50%, 360 participants need to be recruited.

## Results

A total of 1337 students aged between 15 and 20 years from four senior high schools completed the baseline survey of whom 775 students consented to participate in the MoodGYM trial. Table 1 shows gender distribution, mean age, depression, self-efficacy and self-esteem scores and percentage of participants reporting a current need for help in the baseline survey-only group and in the MoodGYM trial group, respectively. Although 775 students consented to participate in the MoodGYM trial, only 707 were enrolled in the trial due to a data entry error.

**Table 1:** Sample characteristics and assessment of differences between the survey group and the MoodGYM trial group

	**Baseline survey only n = 562**				**MoodGYM trial n = 775**				
	***n***	***%***	***mean***	***sd***	***n***	***%***	***mean***	***sd***	***p-value***
*Female gender*	235	41.8			440	56.8			<.001
*Age*			16.78	1.04			16.80	1.00	.63
*Average grade*			3.95	0.78			4.13	0.80	<.001
*Depressive symptoms*			9.81	5.07			11.18	5.96	<.001
*Self-efficacy*			29.32	5.01			29.28	4.61	.88
*Self-esteem*			18.92	3.96			17.99	4.21	<.001
*Current need of help*	51	9.07			130	16.77			<.001

### MoodGYM trial versus survey-only participants

Multivariate analysis of variance and chi square tests were performed to assess differences between the MoodGYM group and the baseline survey-only group in gender, age, average grade, depressive symptoms, self-efficacy, self-esteem and self-reported current need of help. The MoodGYM trial group reported a higher average grade F(1,1285) = 13.90, p < .001, more depressive symptoms F(1,1332) = 19.23, p < .001, and lower self-esteem F(1,1317) = 16.31, p < .001 compared to the survey-only group. There was no significant difference in age and self-efficacy scores. Also, in the MoodGYM trial group there were more females, Pearson’s χ^2^(1) = 29.16, p < .001, and more individuals self-reporting current need of help Pearson’s χ^2^(1) = 16.50, p < .001.

### Testing of assumptions in MoodGYM trial

To test the assumption of statistically independent observations, intra-class correlations (ICCs) were calculated for each of the outcome measures within the first, second and third year at each school. There was no evidence of clusters based on year. Intra-class correlations were 0.03 for depression (CES-D), 0.05 for self-esteem, 0.02 for initial MoodGYM uptake and 0.01 for MoodGYM adherence, supporting the notion of independent observations.

### Attrition

**Figure 1:**
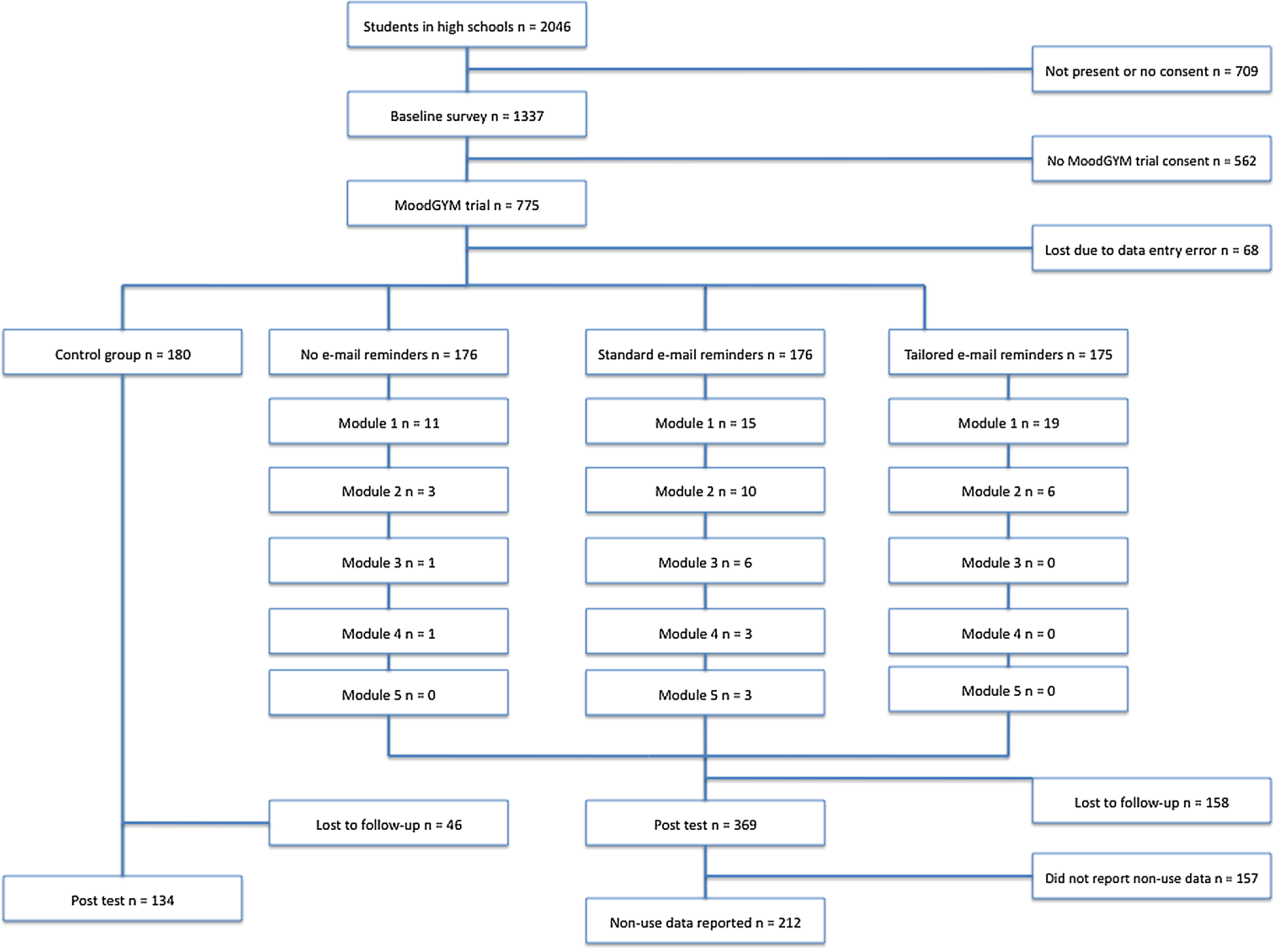
Flow of participants through the trial.

Figure 1 presents the flow of participants through the trial. 8.54% (45/527) of the participants in the intervention groups actually signed on and used MoodGYM. 70.02% (369/527) of participants randomized into an intervention group returned post intervention questionnaires. Of these, 40.23% (212/527) reported post- intervention data regarding non-use.

### Intervention efficacy

Due to the low uptake of the intervention, the users of MoodGYM were collapsed into one group regardless of their initial allocation for the purpose of examining intervention effects. A multivariate analysis of variance was performed, comparing MoodGYM users with complete pre- and post intervention data (n = 42) to non-users (n = 483). Using Pillai's trace testing for differences between the groups on depression or self-esteem, no significant differences was found, F (2,522) = 1.04, p = .36.

To test for possible intervention effects among participants with elevated symptoms the analysis was repeated for participants with a CES-D score above the 16-point cutoff at baseline. Using Pillai's trace testing for differences between the MoodGYM users (n = 19) and non-users (n = 179) with complete pre- and post data, no significant differences were found for depression and self-esteem, F (2,195) = 0.01, p = .99.

### Univariate correlations

As the hypothesized relationships regarding uptake and adherence only concern the intervention groups, the control group will be excluded from further analysis. Table 2 provides an overview of the variable correlations across the MoodGYM trial intervention groups. All variables, except MoodGYM use, are measured at baseline.

**Table 2:** Variable correlations across the MoodGYM intervention groups

	**Gender**	**Age**	**Average grade**	**Current need**	**Depression**	**Self-esteem**	**Self-efficacy**	**MoodGYM use**
Gender	-							
Age	-.09*	-						
n	525							
Average grade	.09*	-.14**	-					
n	509	507						
Current need	.11*	.13**	-.13**	-				
n	521	519	503					
Depression	.20***	.06	-.15***	.57***	-			
n	527	525	509	521				
Self-esteem	-.25***	.00	.24***	-.42***	-.65***	-		
n	524	522	506	518	524			
Self-efficacy	-.13**	-.03	.22***	-.25***	-.40***	.51***	-	
n	521	519	504	515	521	518		
MoodGYM use	.03	-.02	.16***	.04	.07	-.08	-.07	-
n	527	525	509	521	527	524	521	

*p < .05. **p < .01. ***p < .001.

Note. Gender: Higher value represents female. Average grade: Range is 1 to 6. Current need and MoodGYM use: Dichotomous yes/no variables.

### Baseline differences between trial groups

We examined possible differences at baseline in depressive symptoms, self-esteem and self-efficacy between the three intervention groups. Using Pillai’s trace, there were no significant differences across intervention groups in gender, age, average grade, current need of help, depressive symptoms, self-esteem or self-efficacy F(14,998), 1.16, p = .30. The number of participants reporting a current need for help in each intervention group ranged from 24 to 35, χ^2^(2) = 3.02, p = .21.

### Effects of tailored e-mail reminders and current need of help on initial uptake

A hierarchical logistic regression analysis was performed to test for possible effects of tailored e-mail reminders and self-reported current need of help on initial uptake. The overall uptake of MoodGYM across the three intervention groups was unexpectedly low, with only 45 participants (8.54%) entering the program. The predictors were entered blockwise as follows: gender, age, average grade; intervention group affiliation and current need of help; and intervention group affiliation by current need of help. The final model was significant, χ^2^(8) = 20.36, p = .009. However, the only significant predictor of initial uptake was average grade, (Wald statistic = 12.73, p < .001) with an odds ratio of 2.37 (95% CI 1.48-3.80). Intervention group affiliation did not predict initial uptake of MoodGYM, and nor did current need of help or the interaction variable.

### Effect of weekly reminders on adherence

An ordinal regression analysis was performed to test for possible effects of weekly e-mail reminders on adherence. A negative log-log link function was selected because lower values of the outcome variable are more likely to occur. Due to the substantial attrition from the intervention, users persisting from module 2 and onwards were collapsed to form one group in the analysis in order to increase power. As such, adherence was measured as 0 modules, 1 module or 2 or more modules. The overall model was non-significant, χ^2^(1) = 1.92, p = .17, indicating that the weekly reminders did not predict adherence.

### Data regarding reasons for non-usage

Considering the low uptake rate we investigated possible reasons for non-use reported in the group of non-users (n = 662) compared to the MoodGYM users (n = 45). We ran crosstabs testing for between group differences using chi-square tests. As there were no differences in the reports from users versus non-users, the results are presented for the sample as a whole in Table 3.

**Table 3:** Reasons for non-use

***Question***	***n***	***Somewhat or fully disagree***	***Neither disagree nor agree***	***Somewhat or fully agree***
*I did not have access to a computer where I could work undisturbed*	*212*	*157*	*25*	*30*
		*74.1%*	*11.8%*	*14.1%*
*I doubt that such a program can help me*	*210*	*59*	*87*	*64*
		*28.1%*	*41.4%*	*30.5%*
*I forgot about it or could not spare the time*	*208*	*36*	*52*	*120*
		*17.3%*	*25%*	*57.7%*
*I did not have the need for or interest in such a program*	*211*	*78*	*89*	*44*
		*36.9%*	*42.2%*	*20.9%*
*I felt the need to talk to someone, rather than doing this program*	*211*	*83*	*67*	*61*
		*39.3%*	*31.8%*	*28.9%*
*I did not feel sure that I was anonymous*	*211*	*149*	*32*	*30*
		*70.6%*	*15.2%*	*14.2%*

## Discussion

This study aimed to explore the feasibility of disseminating a fully automated self-directed internet-based mental health intervention in senior high schools, and highlights several problems with this procedure. We also aimed to investigate whether automated, tailored e-mails, compared to standardized e-mails, could enhance uptake of the intervention, particularly among those expressing a need for help. The results did not provide evidence to support this idea. Finally, we aimed to explore whether weekly e-mail reminders increased adherence to MoodGYM, but this was not supported by the data.

We found that students consenting to participate in the MoodGYM trial reported more depressive symptoms than those who opted to complete the baseline survey only. Also, students who consented to participating in the trial were more likely to report a current need of help for psychosocial issues. This suggests that those with depression or other psychosocial issues were more open to the intervention than their counterparts. Females were also more likely to volunteer for the trial group, possibly because young women report higher levels of depressive symptoms than men, a finding in line with the literature on gender differences in depression [60].

This study found no support for the prevention hypothesis, that MoodGYM would enhance the participants' self-esteem and reduce risk of depression. Among participants with elevated baseline scores on depression and low self-esteem scores, there was no significant change in the expected directions, thus the treatment hypothesis did not receive support either. Previous research has provided early support for the effectiveness of MoodGYM in reducing depression [24,61] but effects have been variable ranging from small in a universal sample of students in schools [24] to large in an adult telecounselling environment [11]. One explanation for the lack of effect in our study might be that the study does not have sufficient power to yield an effect of a smaller magnitude. Furthermore, very few users proceeded beyond the first two modules, suggesting that the participants received an insufficient dose of the intervention to effect changes in depression or self-esteem.

The hypothesis that individually tailored e-mails would promote the uptake of MoodGYM, particularly among students expressing a current need for support, was not supported. Nor did standardized e-mail prompting enhance initial uptake, compared to providing only the starter e-mail. This contradicts other research reporting an effect of the use of e-mail reminders to increase response rates [39-41]. The only significant predictor of initial uptake was average grade in senior high school, with higher grades predicting initial uptake. The relationship between average grade and use of a mental health intervention is unclear, but it is possible that students who are more conscientious in their school work tend to also be more conscientious in following the study protocol once they have consented.

There could be several explanations for why e-mail tailoring did not enhance initial uptake. Firstly, it is possible that the information session and tailored e-mail content was not able to convey the message in a convincing manner. Recent research indicates that online interventions were the preferred mode of delivery of mental health services in only 16% of a sample of Australian adolescents [62]. We did not set out to investigate the preferred service mode in our study, but some 30% of respondents reported that they doubted the usefulness of the intervention. Previous research has shown that beliefs about treatment are significant predictors of perceived need for treatment [63], and also of adherence to an internet-based depression prevention intervention [64]. Internet-based interventions may be disadvantaged by a lack of knowledge among young people of its possible benefits in the general population, and thus be considered less desirable than alternative treatments. Considering the possible reluctance by young people to undertake online interventions, the e-mail content might be important in promoting the use of MoodGYM. The content used in the current trial was formulated by the research group, and we did not undertake a pilot study to explore responses to the wording of the e-mails. Secondly, there is the possibility that the tailored e-mails initiated help-seeking behaviours that did not include MoodGYM, e.g. seeking counseling from the school nurse. It has been reported that frequency of access of an online health promotion web site (YooMagazine) that focused on mental health literacy, early detection of difficulties and help-seeking was associated with greater off-line help-seeking behaviours in an adolescent sample [65]. Off-line help-seeking was not measured in the current study. Thirdly, we cannot rule out errors of measurement as a possible explanation. For instance, we do not know that students actually received and read the e-mails, even if they were dispatched to the correct addresses. There is the possibility that they were considered spam, and perhaps not even appeared in the student’s inbox. It is also possible that students did not regularly check their e-mail. Short Messaging Service (SMS) should be considered as a means of delivering prompting messages. Finally, the study was not sufficiently powered to detect a small effect of tailoring e-mails. Only 20 to 30 individuals in each intervention group expressed a need for help, and only 45 individuals used MoodGYM across the three intervention groups. Thus, the ability to detect small to moderate effects was limited.

Lack of power may also have interfered with testing the hypothesis that weekly e-mail prompting (standardized or tailored e-mails) would enhance adherence to MoodGYM. Due to the low number of MoodGYM users, and the substantial attrition during the first and second module, we chose to merge the data of users of two or more modules into one group, comparing it to that for users of one module and non-users. Despite these steps taken to increase power, there was no effect of receiving weekly prompts. There is other evidence that reminder e-mails increase adherence in adult community samples [16], but it is uncertain if this is the case with adolescents, or if more extensive support is necessary for this group. Other school-based studies have provided an online intervention as part of the curriculum, undertaken within the school environment and led by a teacher or professional. This includes a study of the MoodGYM intervention employed in this study [24]. This mode of delivery attracted more users than did the current self-directed intervention completed outside school hours, and retention was superior [24,66,67].

Allowing for internet based interventions to be implemented during school hours deals with the most prominent reason for non-use reported in our data, namely that the students could not spare the time or forgot about the programme. It seems that the self-directed nature of this mode of delivery was not suitable for the sample in this study, and the findings are similar to previous research, where there was substantial drop-out in a community sample of adolescents [37]. In a monitored environment, there is less drop-out [23,37]. When attempting a universal delivery of mental health programmes regardless of individual need or risk, completely self-directed interventions might not be an appropriate procedure to engage adolescents, as their motivation and persistence may fluctuate, as suggested by our results. Rather, a structured approach would seem preferable. There is some evidence of the effectiveness of universal depression prevention programmes [24], and pursuing the most appropriate means of implementing such programmes is warranted [68].

The findings challenge the approach of promoting a self-directed intervention among senior high school students. Possibly, alternative initial strategies of increasing interest, involvement or commitment in students might have increased uptake. However, the current study shows that tailored feedback adapted to the participants’ level of depression, self-esteem and self-efficacy failed to markedly influence uptake.

## Conclusion

In conclusion, this study revealed the substantial challenges associated with implementing a fully self-directed intervention in an adolescent sample. Universal school internet-based interventions may, despite our findings, have benefits since they have the potential to minimize barriers to help seeking including stigma and a belief in self-reliance. From a preventive public health perspective these benefits should not be underestimated. However, guided interventions rather than purely self-guided approaches may be a more suitable model for the delivery of mental health interventions to adolescents.

### Limitations

This study has several methodological limitations that should be taken into account when interpreting the results.

First, since MoodGYM is an open access site, students might have accessed it via a separate user name and password rather than using those sent by the research team. Thus, the number of actual MoodGYM users remains unknown, as we only are aware of those applying their assigned user names. High school students are skilled and frequent internet users. We observed during school visits that they accessed the MoodGYM web site as the presenter was talking. These limitations could compromise the results, as participants registered as non-users could have registered beyond the research trial.

Another limitation is the selection of schools, which was not carried out randomly. Some schools agreed to participate in the MoodGYM trial after invitation whereas others declined. We are not aware of the motivation for the schools to participate. Possibly, some of the participating schools were experiencing higher levels of psychological difficulties among their students, or had limited resources to deal with mental health issues. Such factors could influence the school´s decision to participate, and could affect the results of the study.

## Competing interests

The authors declare that they have no competing interests.

## Authors’ contributions

KMG, KW and MRE participated in the design of the study, interpretation of results and helped drafting the manuscript. KL and HCBV carried out the data collection, statistical analysis and drafting of the manuscript. All authors read and approve the final manuscript.

## Pre-publication history

The pre-publication history for this paper can be accessed here:

http://www.biomedcentral.com/1471-244X/14/14/prepub
